# National survey of protective eye care practices in the critically ill

**DOI:** 10.1186/cc9958

**Published:** 2011-03-11

**Authors:** R Kam, S Haldar, E Papamichael, K Pearce, M Hayes, N Joshi

**Affiliations:** 1 Imperial College London, UK; 2 North West London Hospitals NHS Trust, London, UK; 3 Watford Hospital, London, UK; 4 University of Nottingham, UK; 5 Chelsea and Westminster Hospital, London, UK

## Introduction

Critically ill patients with inadequate lid closure are susceptible to developing exposure keratopathy. Protective measures can prevent this and reduce the risk of subsequent microbial keratitis and irreversible visual loss. Of two published surveys [1,2], one was carried out before there was any evidence-based research in this area and the other did not address which methods were in use in ICUs. This study aims to describe the current eye care methods used in ICUs in England, the perceived incidence of eye complications, their nature and the effectiveness of protocols in use.

## Methods

A team of researchers telephoned all general ICUs and other specialty critical care units in England caring for sedated, ventilated patients and asked a supervising nurse questions from a questionnaire piloted earlier in London.

## Results

Two hundred and seventeen out of 267 ICUs (81%) participated. One hundred and thirty out of 217 (60%) ICUs had an eye care protocol. Sixty-six per cent of units with protocols assessed lid closure compared with 65% of those without. Geliperm application was the most common protective therapy (106 units, 49%), followed by Lacrilube (76 units, 35%). Most ICUs used a combination of methods. The total estimated incidence of ocular complications in the last year was 502. The most recent complications witnessed included corneal ulceration (23 cases), microbial keratitis (11) and chemosis (23). Cases of severe visual loss were caused by anterior ischaemic optic neuropathy following prone positioning (two cases) and microbial keratitis in a patient's only functioning eye.

## Conclusions

There is a need for protocols that encourage proper eyelid position assessment, effective protection of the ocular surface and referral to ophthalmologists in the event of any complications or any loss of corneal clarity.
